# A patient survey indicates quality of life and progression-free survival as equally important outcome measures in multiple myeloma clinical trials

**DOI:** 10.1007/s00432-023-05137-8

**Published:** 2023-07-19

**Authors:** Anna Fleischer, Larissa Zapf, Johannes Allgaier, Karin Jordan, Götz Gelbrich, Rüdiger Pryss, Johannes Schobel, Max Bittrich, Hermann Einsele, Martin Kortüm, Imad Maatouk, Niels Weinhold, Leo Rasche

**Affiliations:** 1 Department of Internal Medicine II, University Hospital Würzburg, Oberdürrbacherstr. 6, 97080 Würzburg, Germany; 2 Institute of Biometry and Epidemiology, University Hospital of Würzburg, Würzburg, Germany; 3 Department of Hematology, Oncology and Palliative Medicine, Ernst Von Bergmann Hospital, Potsdam, Germany; 4 Department of Medicine V, Hematology, Oncology and Rheumatology, University of Heidelberg, Heidelberg, Germany

**Keywords:** Multiple myeloma, Quality of life, Progression-free survival, Endpoint measure, Patient involvement, Lenalidomide maintenance therapy

A key challenge in cancer therapy is to balance potential survival benefit against treatment-related toxicity and subsequent impairment of Quality of Life (QoL). The oncologist’s role is not only to deliver the best quality anticancer treatment but also to consider the impact of the disease and treatment on each patient (Jordan et al. 2018). In Multiple Myeloma (MM) patients are usually continuously treated for several years. Continuous therapy, however, constantly exposes patients to displeasing side effects (Jordan et al. 2014). QoL in MM patients deteriorates with each subsequent line of therapy (Engelhardt et al. 2021). QoL measurements have been implemented as a secondary endpoint in almost all recent MM trials. Yet, it is important to note that the patients’ preference on a survival benefit versus a potentially impaired QoL has yet to be studied in MM, e.g. it is unknown whether patients would accept reduced survival for better QoL or vice-versa.

Maintenance therapy with lenalidomide (LEN) is a scenario in which this preference appears highly relevant (Richardson et al. 2022). On the one hand, two meta-analyses confirmed a progression-free survival (PFS) benefit in patients treated with LEN until progression of roughly 24 months compared to placebo or observation (McCarthy et al. 2017). On the other hand, a number of LEN-related side effects such as thromboembolism, diarrhea, peripheral neuropathy, constipation, and muscle pain were frequently observed (Pawlyn et al. 2014). Further, the incidence of second primary malignancies (SPMs) was reported to be three-fold higher for patients treated with LEN (Holstein et al. 2017). These negative effects, which mainly included grade 1 toxicities, sum up to a clear, yet undetermined deficit in QoL during LEN maintenance.

To address the question whether continuous LEN matches with the patient's preference on maintenance therapy, we analyzed patient reported outcome measures on maintenance therapy and related clinical endpoints in patients with MM. We actively involved MM patients to develop an online survey of 205 questions tailored especially to the needs of patients with MM under LEN maintenance therapy. The survey contained two validated questionnaires (EORTC, QoL questionnaires C30 and My20) on QoL and a set of additional questions pertaining to LEN toxicity and tolerability, which we developed together with a focus group of patients from the University Hospital Würzburg (Supplemental Table 1). To directly address the patient’s preference, we included an additional questionnaire that asked patients, whether they would choose a shortened time of PFS in favor of an increased QoL (Table 1). We distributed the online survey with the help of patient advocacy groups for MM patients in Germany. Patients who were interested in participating in our survey anonymously logged in to our public homepage and answered the questions. The survey was open for a timeframe of 50 days.

**Table 1:** Patient preferences regarding outcome measures (PFS and QoL)

	Preference			All patients
	Long PFS	High QoL	None	
Number of patients	92	81	21	194
Advanced treatment line				
Yes	21 (11%)	32 (16%)	11 (6%)	64 (33%)
No	71 (37%)	49 (25%)	10 (5%)	130 (67%)
Fisher exact test statistic value < 0.021	The result is significant at *p* < 0.05			
Number of patients	90	79	21	190
Tendency to hand over responsibility to physicians				
Yes	31 (16%)	14 (7%)	7 (4%)	52 (27%)
No	59 (31%)	65 (34%)	15 (8%)	138 (73%)
Fisher exact test statistic value < 0.0153	The result is significant at *p* < 0.05			
Number of patients	92	76	18	186
Diarrhoea				
Mild or none	79 (42%)	65 (35%)	16 (9%)	160 (86%)
Severe or very severe	13 (7%)	11 (6%)	2 (1%)	26 (14%)
Fisher exact test statistic value = 1	The result is *not* significant at *p* < 0.05			
Number of patients	90	75	19	184
Nausea				
Mild or none	87 (47%)	72 (39%)	19 (10%)	178 (97%)
Severe or very severe	3 (1.5%)	3 (1.5%)	0	6 (3%)
Fisher exact test statistic value = 1	The result is *not* significant at *p* < 0.05			
Number of patients	90	75	18	183
Constipation				
Mild or none	84 (46%)	69 (38%)	18 (9%)	171 (93%)
Severe or very severe	6 (3%)	6 (3%)	0	12 (6%)
Fisher exact test statistic value = 0.77	The result is *not* significant at *p* < 0.05			
Number of patients	90	76	19	185
Fatigue				
Mild or none	70 (38%)	60 (32%)	15 (8%)	145 (78%)
Severe or very severe	20 (11%)	16 (9%)	4 (2%)	40 (22%)
Fisher exact test statistic value = 1	The result is *not* significant at *p* < 0.05			
Number of patients	92	76	19	187
Fever				
Yes	2 (1%)	4 (2%)	0	6 (3%)
No	90 (48%)	72 (39%)	19 (10%)	181 (97%)
Fisher exact test statistic value = 0.41	The result is *not* significant at *p* < 0.05			
Number of patients	90	73	19	182
Upper airway infection				
Mild or none	90 (49%)	70 (38%)	19 (10%)	179 (96%)
Severe or very severe	0	3 (2%)	0	3 (2%)
Fisher exact test statistic value = 0.09	The result is *not* significant at *p* < 0.05			
Number of patients	90	76	19	185
Pulmonary infection				
Mild or none	85 (46%)	74 (40%)	19 (10%)	178 (96%)
Severe or very severe	5 (3%)	2 (1%)	0	7 (4%)
Fisher exact test statistic value = 0.46	The result is *not* significant at *p* < 0.05			
Number of patients	90	75	19	184
Dyspnea				
Mild or none	81 (44%)	69 (38%)	18 (9%)	168 (91%)
Severe or very severe	9 (5%)	6 (3%)	1 (1%)	16 (9%)
Fisher exact test statistic value = 0.79	The result is *not* significant at *p* < 0.05			
Number of patients	91	76	19	186
Vertigo				
Mild or none	85 (46%)	72 (39%)	19 (10%)	176 (95%)
Severe or very severe	6 (3%)	4 (2%)	0	10 (5%)
Fisher exact test statistic value = 0.76	The result is *not* significant at *p* < 0.05			
Number of patients	91	75	19	185
Sensory peripheral neuropathy				
Mild or none	83 (45%)	66 (36%)	18 (9%)	167 (90%)
Severe or very severe	8 (4%)	9 (5%)	1 (1%)	18 (10%)
Fisher exact test statistic value = 0.61	The result is *not* significant at *p* < 0.05			
Number of patients	89	75	19	183
Secondary malignancy				
Yes	6 (3%)	3 (2%)	1 (1%)	10 (6%)
No	83 (45%)	72 (39%)	18 (10%)	173 (94%)
Fisher exact test statistic value = 0.51	The result is *not* significant at *p* < 0.05			
Number of patients	92	74	18	184
General muscular weakness				
Yes	35 (19%)	32 (17%)	9 (5%)	76 (41%)
No	57 (31%)	42 (23%)	9 (5%)	108 (59%)
Fisher exact test statistic value = 0.53	The result is *not* significant at *p* < 0.05			
Number of patients	89	76	19	184
Muscle cramps				
Mild or none	74 (40%)	62 (34%)	17 (9%)	153 (83%)
Severe or very severe	15 (8%)	14 (8%)	2 (1%)	31 (17%)
Number of patients	91	76	19	186
Thrombosis/thromboembolism				
Yes	14 (8%)	11 (6%)	4 (2%)	29 (16%)
No	77 (41%)	65 (35%)	15 (8%)	157 (84%)
Fisher exact test statistic value = 1	The result is *not* significant at *p* < 0.05			
Number of patients	88	78	21	187
Back pain				
Yes	10 (5%)	4 (2%)	3 (1%)	17 (9%)
No	78 (42%)	74 (40%)	18 (9%)	170 (91%)
Fisher exact test statistic value = 0.17	The result is *not* significant at *p* < 0.05			
Number of patients	88	79	21	188
Hip pain				
Yes	87 (46%)	78 (41%)	21 (11%)	186 (99%)
No	1 (1%)	1 (1%)	0	2 (1%)
Fisher exact test statistic value = 1	The result is *not* significant at *p* < 0.05			
Number of patients	91	80	20	191
Arm or shoulder pain				
Yes	46 (24%)	51 (27%)	8 (4%)	105 (55%)
No	45 (24%)	29 (15%)	12 (63%)	86 (45%)
Fisher exact test statistic value = 0.09	The result is *not* significant at *p* < 0.05			
Number of patients	89	78	21	188
Chest pain				
Yes	24 (13%)	27 (14%)	9 (5%)	60 (32%)
No	65 (35%)	51 (27%)	12 (6%)	128 (68%)
Fisher exact test statistic value = 0.32	The result is *not* significant at *p* < 0.05			
Number of patients	91	81	21	193
Dry mouth				
Yes	50 (26%)	45 (23%)	12 (6%)	107 (55%)
No	41 (21%)	36 (19%)	9 (5%)	86 (45%)
Fisher exact test statistic value = 1	The result is *not* significant at *p* < 0.05			
Number of patients	90	78	21	189
Hair loss				
Yes	20 (11%)	20 (11%)	6 (3%)	46 (24%)
No	70 (37%)	58 (31%)	15 (8%)	143 (76%)
Fisher exact test statistic value = 0.72	The result is *not* significant at *p* < 0.05			
Number of patients	91	81	21	193
Heartburn				
Yes	41 (21%)	32 (17%)	12 (6%)	85 (44%)
No	50 (26%)	49 (25%)	9 (5%)	108 (56%)
Fisher exact test statistic value = 0.54	The result is *not* significant at *p* < 0.05			
Number of patients	86	78	21	185
Lenalidomide maintenance therapy				
At the time of the survey	68 (36%)	48 (26%)	18 (10%)	134 (72%)
Before the time of the survey	18 (10%)	30 (16%)	3 (2%)	51 (28%)
Fisher exact test statistic value = 0.0164	The result is significant at *p* < 0.05			

Of 194 patients with MM who answered this question, an unexpected high number of 81 (42%) subjects were willing to accept a shorter PFS for better QoL. On the other hand, 92 (47%) preferred a longer PFS at the cost of reduced QoL. Twenty-one patients (11%) indicated to be undecided.

**Figure:**
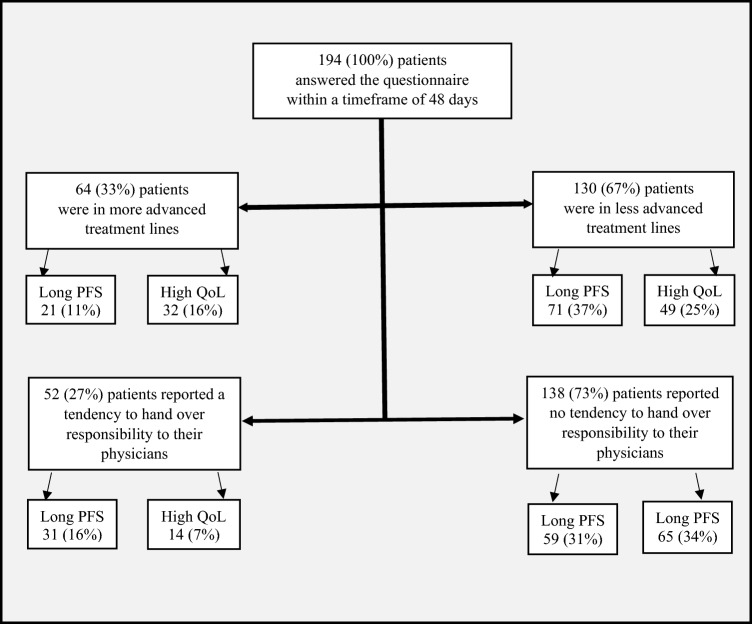
Patient flow

We next addressed the question whether specific features were associated with the two main groups (“in favor QoL” vs “in favor PFS”) (Sacristán et al. 2016). Patients who belonged to the “in favor QoL”-group tended to be in more advanced treatment lines when compared to the “in favor PFS-group” (*P* = 0.0001; Fisher test, not corrected for multiple testing). Those patients who had received LEN maintenance therapy before the time of the survey and whose LEN therapy had been terminated before the time the survey was undertaken, were significantly more likely to belong to the “in favor QoL”-group. Patients who preferred PFS were found to generally be more likely to hand over responsibility to their physicians (*P* = 0.01; Fisher test). No associations were found for other disease specific conditions including pain, gastrointestinal symptoms, fatigue or infection (Table 1). Of note, we did not find differences between severe or very severe side effects being associated with one of the two groups. It is important to take into consideration, that these results were gathered using an anonymous web-based questionnaire, and patients’ preferences on possible outcome measures in myeloma may change over time during the course of treatment and have yet to be determined. Despite these limitations, we conclude that QoL constitutes the central outcome measure for roughly half of our patients.

Planning a new generation of clinical trials requires active involvement of patients to value their preferences concerning study endpoints (Mohyuddin et al. 2022; Auclair et al. 2022; Mols et al. 2012). It is important to consider the patient’s perspective to adapt study design and endpoints to the needs of the patients. This procedure may add a new dimension to traditional outcome measures specifically regarding the primary endpoint. While capturing changes in QoL has become standard in clinical trials, it remains difficult for both patients and treating physicians to envision the trade-off between survival outcome and QoL and to use this information for shared decision-making. In our opinion, statistically significant results alone are barely helpful. As an alternative approach, we propose to provide a trade-off between PFS and QoL presented in terms of likelihood rather than statistical significance. For instance, for an individual patient treated in arm A of a given study, the likelihood of being progression-free at 3 years from treatment may be 80% and QoL 70%, whereas in arm B PFS likelihood is 60% and QoL 90%.

In conclusion, our data strongly suggest that future studies in this setting should include PFS and QoL measures as co-primary endpoints to account for the heterogeneity in patients’ preferences and to collect the information necessary for shared decision-making in future patients. The results of our study accentuate significant differences in patients’ preferences, thus underlining the importance of assessing individual patient needs in determining the endpoints of further research.

### Supplementary Information

Below is the link to the electronic supplementary material.

Supplementary file1 (DOCX 21 KB)

## Data Availability

The data is available upon request.
