# Supplementary material for: A patient survey indicates quality of life and progression-free survival as equally important outcome measures in multiple myeloma clinical trials
Source: J Cancer Res Clin Oncol. 2023 Jul 19;149(14):12897–902. doi: 10.1007/s00432-023-05137-8 (PMC10587328; doi:10.1007/s00432-023-05137-8)
Supplement: Supplementary file 1 — Supplementary file1 (DOCX 21 KB) [file 432_2023_5137_MOESM1_ESM.docx]

***Supplemental table 1***

| Would you like to have a say in the determination of the duration of your lenalidomide treatment, or would you prefer your treating physician to make this decision for you? | 1 | Yes, I would want to have a say. |
| --- | --- | --- |
|  | 2 | No, I would prefer my treating physician to make this decision for me. |
|  |  |  |
| How much would it bother you, to have to take medication in tablet form every day – possibly for years. | 1 | It would bother me very much. |
|  | 2 | It would bother me a little bit. |
|  | 3 | It would hardly bother me at all. |
|  | 4 | It would not bother me at all. |
|  |  |  |
| Would you be more willing to take medication in tablet form regularly, if you knew, that possible side-effects and effects on your well-being as a whole would be registred regularly  e.g. by means of an app and registred by your treating physicians?  The impact of such an app on my willingness, to take tablets regularly would be: | 1 | very big |
|  | 2 | big |
|  | 3 | small |
|  | 4 | no impact at all |
|  |  |  |
| **How important are the following questions for you?** |  |  |
| Which side-effects should I expect? | 1 | very important |
|  | 2 | important |
|  | 3 | less important |
|  | 4 | not important at all |
|  |  |  |
| How often do I have to come to ambulatory controls? | 1 | very important |
|  | 2 | important |
|  | 3 | less important |
|  | 4 | not important at all |
|  |  |  |
| How many days will I spend at the hospital? | 1 | very important |
|  | 2 | important |
|  | 3 | less important |
|  | 4 | not important at all |
|  |  |  |
| How high are my personal financial costs caused by my medication? | 1 | very important |
|  | 2 | important |
|  | 3 | less important |
|  | 4 | not important at all |
|  |  |  |
| How high are the financial costs of the medication on the healtcare system? | 1 | very important |
|  | 2 | important |
|  | 3 | less important |
|  | 4 | not important at all |
|  |  |  |
| Does the medication have an impact on my quality of life? | 1 | very important |
|  | 2 | important |
|  | 3 | less important |
|  | 4 | not important at all |
|  |  |  |
| Will the medication elongate the time until disease progression (time without clinical signs of disease)? | 1 | very important |
|  | 2 | important |
|  | 3 | less important |
|  | 4 | not important at all |
|  |  |  |
| Will the medication increase my survival time? | 1 | very important |
|  | 2 | important |
|  | 3 | less important |
|  | 4 | not important at all |
|  |  |  |
| Which further information would you like to get concerning your treatment? |  | open textfield |
|  |  |  |
| Which role should quality of life play in the decision, when to end a therapy? | 1 | Quality of life should play no role at all. |
|  | 2 | 2 |
|  | 3 | 3 |
|  | 4 | 4 |
|  | 5 | 5 |
|  | 6 | 6 |
|  | 7 | 7 |
|  | 8 | 8 |
|  | 9 | 9 |
|  | 10 | Quality of life should play a very strong role. |
|  |  |  |
| For the decision, when a therapy is terminated, side-effects should … | 1 | … not play any role at all. |
|  | 2 | 2 |
|  | 3 | 3 |
|  | 4 | 4 |
|  | 5 | 5 |
|  | 6 | 6 |
|  | 7 | 7 |
|  | 8 | 8 |
|  | 9 | 9 |
|  | 10 | … play a decisive roll. |
|  |  |  |
| For the decision, when a therapy is terminated, the time of progression free survival until relapse of Multiple Myeloma should … | 1 | … not play any role at all |
|  | 2 | 2 |
|  | 3 | 3 |
|  | 4 | 4 |
|  | 5 | 5 |
|  | 6 | 6 |
|  | 7 | 7 |
|  | 8 | 8 |
|  | 9 | 9 |
|  | 10 | … play a decisive role |
|  |  |  |
| For the decision, when a therapy is terminated, the time of overall survival (until death) should … | 1 | … not play any role at all |
|  | 2 | 2 |
|  | 3 | 3 |
|  | 4 | 4 |
|  | 5 | 5 |
|  | 6 | 6 |
|  | 7 | 7 |
|  | 8 | 8 |
|  | 9 | 9 |
|  | 10 | … play a decisive role |
|  |  |  |
| Which further aspects should be taken into consideration? |  | open textfield |
|  |  |  |
| When the termination of a drug leads to a markedly improved quality of life, I am willing to accept an earlier disease relapse. | 1 | I agree 100%. |
|  | 2 | I agree, but without certainty. |
|  | 3 | I disagree, but without certainty. |
|  | 4 | I disagree 100%. |
|  | 5 | I cannot answer this question or I do not like to answer this question. |
|  |  |  |
| When the termination of a drug leads to a markedly improved quality of life, I am willing to accept a shortened time of overall survival.  (earlier death) | 1 | I agree 100%. |
|  | 2 | I agree, but without certainty. |
|  | 3 | I disagree, but without certainty. |
|  | 4 | I disagree 100%. |
|  | 5 | I cannot answer this question or I don’t like to answer this question. |
|  |  |  |
| Have you relapsed once or more than once? | 1 | yes |
|  | 2 | no |
|  |  |  |
| If yes: How did your quality of life change since your second relapse – has it improved, stayed the same or deteriorated compared to your first relapse? | 1 | It got better. |
|  | 2 | Nothing changed. |
|  | 3 | It got worse. |
|  | 4 | I cannot or do not want to answer this question. |
|  |  |  |
| How hard was it for you to answer this question? | 1 | very hard |
|  | 2 | hard |
|  | 3 | neither easy nor hard |
|  | 4 | relatively easy |
|  | 5 | very easy |
|  |  |  |
| Was it emotionally stressful for you to answer these questions? | 1 | It was very stressful. |
|  | 2 | It was a little stressful. |
|  | 3 | It was hardly stressful at all. |
|  | 4 | It was not stressful at all. |
|  |  |  |
| You can add personal remarks on this questionnaire here |  | open textfield |
|  |  |  |
| Have you ever received lenalidomide? | 1 | No |
|  | 2 | Yes, I am taking lenalidomide at the moment. |
|  | 3 | Yes, I used to take lenalidomide in the past, but not any more. |
|  |  |  |
| How hard was it for you to answer the questions on side effects of lenalidomide according to CTCAE-criteria? | 1 | It was easy. |
|  | 2 | It was neither easy nor hard. |
|  | 3 | It was hard. |
|  |  |  |
| If it was hard for you to answer the questions on side effects of lenalidomide according to CTCAE-criteria – why was it hard for you? |  | open textfield |
|  |  |  |
| How high would the likelihood of severe side effects have to be for you to stop lenalidomide maintenance therapy?  Please insert the number (percentage), you consider appropriate here. |  | number (%) |
|  |  |  |
| By how many percent would your quality of life have to worsen for you to terminate lenalidomide maintenance therapy? |  | number (%) |
|  |  |  |
| How much is a day in full health worth for you compared to a day of severe side effects or disease symptoms?  To answer this question, please complete the following sentence with the number, which comes to your mind. |  | „For me, one day in full health is worth as much as ________________ days with side effects or disease symptoms of high or very high severity.“ |
|  |  |  |
| How long did it take you to fill in the My20-questionnaire? | 1 | < 2 minutes |
|  | 2 | 2-5 minutes |
|  | 3 | > 5 minutes |
|  | 4 | > 10 minutes |
|  |  |  |
| Do you think, it makes sense for patients with Multiple Myeloma to answer the My20-questionnaire? | 1 | yes |
|  | 2 | no |
|  |  |  |
| How often can patients under lenalidomide maintenance therapy be asked, to fill the My20 and the C30 questionnaire, to gain longitudinal data on quality of life? | 1 | daily |
|  | 2 | 1x per week |
|  | 3 | 1x per month |
|  | 4 | every 3 months |
|  |  |  |
| How hard was it for you, to answer the questions on side effects of lenalidomide according to the CTCAE-criteria? | 1 | very easy |
|  | 2 | middle |
|  | 3 | hard |
|  |  |  |
| If it was hard for you, which difficulties did you have? | open_textfield | open_textfieldd |
|  |  |  |
| Did the questionnaire you just answered cover all side-effects of lenalidomide maintenance therapy? | 1 | yes |
|  | 2 | no |
|  |  |  |
| If not, which side-effects of lenalidomide are missing? | open_textfield | open_textfieldd |
|  |  |  |
| How often can patients be asked in future trials, to answer questions on the side-effect profile of lenalidomide (e.g. via smartphone, telephone or computer)? | 1 | daily |
|  | 2 | 1x per week |
|  | 3 | 1x per month |
|  | 4 | every 3 months |
|  |  |  |
| If you had the choice – what would you prefer? | 1 | option A |
|  | 2 | option B |
|  | 3 | Both options should be available. |
